# Biocatalytic Resolution of Enantiomeric Mixtures of 1-Aminoethanephosphonic Acid

**DOI:** 10.3390/molecules16075896

**Published:** 2011-07-14

**Authors:** Małgorzata Brzezińska-Rodak, Magdalena Klimek-Ochab, Ewa Żymańczyk-Duda, Paweł Kafarski

**Affiliations:** Department of Bioorganic Chemistry, Faculty of Chemistry, Wroclaw University of Technology, Wybrzeże Wyspiańskiego 27, 50-370 Wroclaw, Poland

**Keywords:** aminophosphonic acid, fungi, resolution of racemic mixture

## Abstract

Several fungal strains, namely *Bauveria bassiana, Cuninghamella echinulata, Aspergillus fumigatus, Penicillium crustosum* and *Cladosporium herbarum*, were used as biocatalysts to resolve racemic mixtures of 1-aminoethanephosphonic acid using L/D amino acid oxidase activity. The course of reaction was analyzed by ^31^P-NMR in the presence of cyclodextrin used as chiral discriminating agent. The best result (42% e.e of *R*-isomer) was obtained with a strain of *Cuninghamella echinulata.*

## 1. Introduction

Aminophosphonic acids and their analogues are an important class of organophosphorous compounds, mostly because of their varied biological activity [1]. The application of aminophosphonates range from agriculture to medicine, as exemplified by the herbicides glyphosate and phosphiniothricin, the antibacterial alafosfalin, the antihypertensive fosinopril or the antiosteoporetic bisphosphonates [2,3,4,5,6,7,8,9,10]. Aminophosphonic acids, being considered as structural analogues of amino acids, exhibit inhibitory activity against different enzymes, especially towards proteinases such as HIV protease, thrombin, aminopeptidases, and human collagenase [11,12]. 

Various synthetic protocols have been described for the synthesis of α-aminophosphonates, including a plethora of “green” procedures [8,13,14,15,16] and they are thus now readily available in gram quantities as racemates. However, the preparation of enantiomerically pure forms of these compounds, still constitutes a challenging issue and most of the methods for their preparation are cumbersome and of low efficiency. 

The biocatalytic resolution of racemic molecules has attracted the interest of chemists for several decades [17,18,19,20]. Microbial biocatalysts in the form of purified enzymes or whole microbial cells have been successfully used for synthesis of optically pure organophosphonate derivatives [21,22,23,24,25,26,27,28,29], showing the utility of biocatalysis in this branch of chemistry. Surprisingly, bioconversion is scarcely used to obtain optically pure aminophosphonic compounds [30,31,32,33,34,35]. There is only one literature note discussing the application of *Candida antarctica* lipase for the enantioselective acylation of 1-and 2-aminoalkanephosphonates [36]. Consequently, the development of new biocatalytic methods for the obtaining of these acids really is a not fully explored area of research. 

Here we report a simple strategy of enantioselective oxidation of 1-aminoethanephosphonic acid (a warhead of the antibiotic alafosfalin) using whole cells of *fungi imperfecti*. Resolution of the racemic mixture of this acid provided its desired L-and D-isomers with moderate, although satisfactory enantiomeric excesses.

## 2. Results and Discussion

Because of the future applicability, there is still a need for the development of effective methods for preparation of α-aminophosphonic acids in their enantiomerically pure forms. Many approaches have been undertaken to reach this goal, with the most successful being resolutions of racemic mixtures of aminophosphonate esters by application of the derivatives of tartaric acid [37,38]. Several other protocols for asymmetric synthesis of these compounds have also been described although these methods are considered as less effective and cumbersome [16]. 

Biocatalysis is a very attractive alternative for chemical asymmetric synthesis because it is, in the most cases, simple and economic. The biocatalyst (pure enzymes or/and whole microbial cells) are more and more frequently used in chemical, especially asymmetric synthesis, leading to structurally diverse, optically pure compounds. 

In previous works we have employed various strains of fungi for the synthesis of P-chiral hydroxyphosphonates [21] and for the oxidative resolution of racemic mixtures of diethyl 1-hydroxy-phenylmethanephosphonate [39]. The success of these reactions was an inspiration for the use of whole cells of microorganisms for the resolution of racemic mixtures of 1-aminoethanephosphonic acid. This molecule was chosen as a standard substrate, because of its simple structure and mentioned application and, what is also important, because of the available analytical methods for the identification and absolute configuration assignment. 

**Scheme 1:**
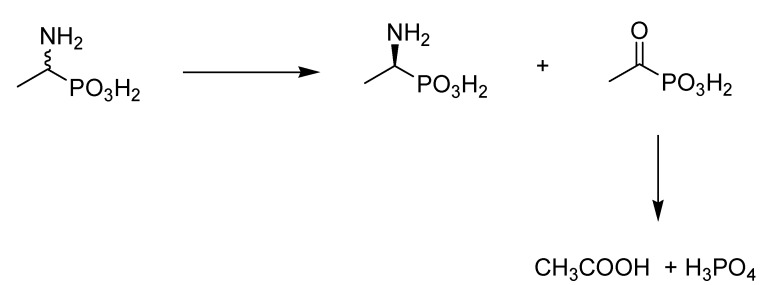
Enantioselective degradation of 1-aminoethanephosphonic acid by *Cuninghamella echinulata*.

The phosphonic analogue of alanine is not a physiological substrate for fungi, therefore in some cases the incubation of the biocatalysts cells (before bioconversion) under starvation conditions was required to force them to utilize such a compound as an exogenous source of biogenic phosphorus –a crucial element for viability of the cells. Thus, our strategy relayed on the premise that some fungi are known for their capacity to degrade slowly aminophosphonates [40,41]. The pathway of this utilization most likely proceeds *via* oxidative conversion of aminophosphonate to ketophosphonate (Scheme 1). The ketophosphonate, in turn, is chemically unstable and undergoes chemical degradation to phosphate and acetic acid. The question, which remained was if this process is stereoselective. 

Five strains of *fungi imperfecti* have been tested in this work, namely *Cuninghamella echinulata*, *Aspergillus fumigatus*, *Beauveria bassiana*, *Penicillium crustosum*, *Cladosporium herbarum*. In order to obtain the product of desired configuration, the biomass was prepared with or without the starvation period following routine cell cultivation. Results reached with *C. echinulata*, after the inspection of the ^31^P-NMR spectra of the crude reaction mixture, showed the presence of phosphate as the only second phosphorus product, supporting our speculation. Thus, enrichment of 1-aminoethanephosphonic acid with the *R*-isomer (42% e.e) after two days of biotransformation with fresh cells and without any starvation period was obtained. Quite interestingly, this fungus preferably decomposed *S-*enantiomer, which corresponds to unnatural D-configuration. The same enantioselectivity was exhibited by *C. echinulata*, when it was used under starvation conditions (Table 1). Also a drop in enantiomeric excess was observed as the time of cultivation of the microorganism in the presence of phosphonic analogue of alanine was increased.

The inversion of the absolute configuration seems to be quite general phenomenon (Table 1). A possible explanation of this phenomenon is that, that bacterial amino acid oxidases are either constitutive or inducible and their activity most likely depends on the time of incubation of the cell and aminophosphonate. Moreover, the protein heterogeneity (e.g., enantiospecificity) often results from the coexistence of interconvertible isomeric forms of the monomeric enzyme, which also can be an explanation of the disscused phenomenon. 

**Table 1:** Biooxidative resolution of 1-aminoethanephosphonic acid.

Microorganism	fresh cells: e.e values^1^/configuration					cells after starvation: e.e values/configuration				
**Day of transformation**	**1 **	**2**	**3**	**4**	**5**	**1**	**2**	**3**	**4**	**5**
*Cuninghamella echinulata*	28% *R*	42% *R*	25% *R*	25% *R*	13% *R*	26% *R*	21% *R*	13% *R*	12% *R*	9% *R*
*Aspergillus fumigatus*	9% *S*	10% *S*	11% *S*	0	11% *S*	25% *S*	13% *R*	6% *R*	12% *R*	13% *R*
*Beauveria bassiana*	13% *R*	4% *S*^2^	10% *R*	6% *R*	6% *R*	2% *S*^2^	8% *R*	2% *S*^2^	5% *S*	6% *R*
*Penicillium crustosum*	14% *R*	14% *R*	3% *R*^2^	2% *R*^2^	6% *S*	9% *R*	8% *R*	7% *S*	6% *S*	0
*Cladosporium herbarum*	0	0	6% *S*	18% *S*	6% *R*	0	0	4% *R*	12% *S*	3% *R*^2^

^1^ average value from three runs with statistic error = 3%, ^2^ results included in the margin of error.

The enrichment of product of *S* configuration was observed, when the biotransformation was carried out with whole cells of *Aspergillus fumigatus*. The highest enantiomeric excess was obtained after only one day of biotransformation with starved cells (25% e.e; Table 1). Quite interestingly, cultivation of the fungi for longer periods resulted in a reversal of enantioselectivity. Fresh cells of *A. fumigatus* demonstrated poorer activity, yielding predominantly the *S* enantiomer. The enantiomeric excess in these experiments was around 10% (Table 1). 

The application of other fungal cells was less effective, but still supports the idea that microbial cells are able to degrade aminophosphonates enantioselectively allowing tapping this process as a mean to prepare optically pure phosphonic acid. However, the presented results are of preliminary nature and intensive search for more efficient microorganisms alongside with optimization of the conditions of this process are required. 

**Figure 1:**
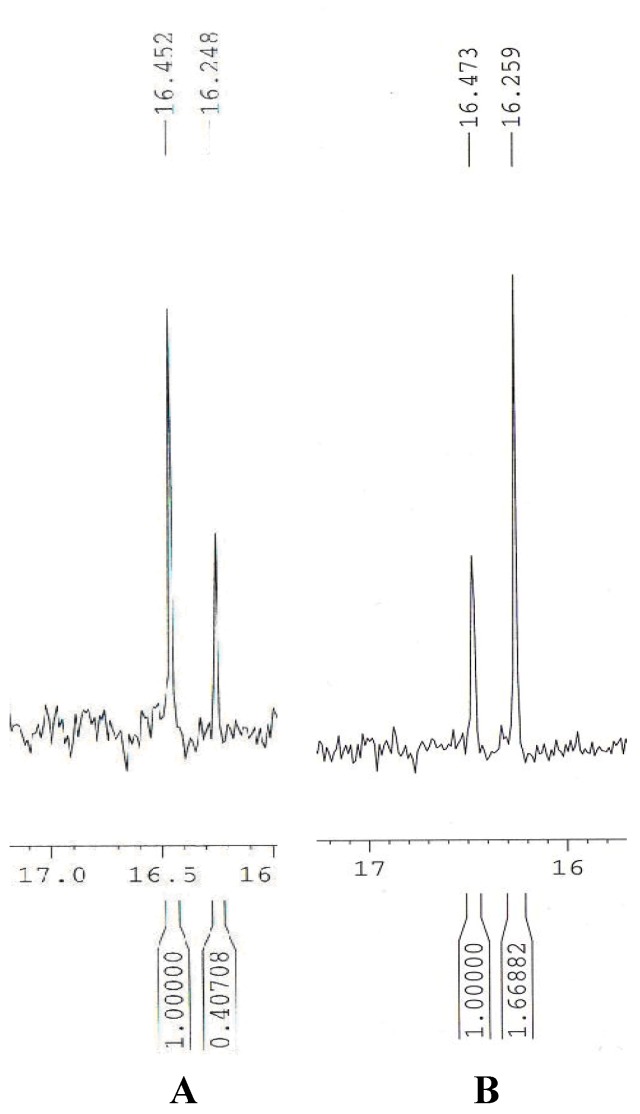
^31^P-NMR spectra of biotransformation products recorded with cyclodextrin: **A**, fresh cells of *C.echinulata*, second day of biotransformation, predominant enantiomer defined as *R* isomer; **B**, cells of *A. fumigatus* preincubated under starvation conditions, first day of biotransformation, predominant enantiomer defined as *S* isomer.

## 3. Experimental

### 3.1. General

All the chemicals and solvents were purchased from commercial sources. NMR spectra were recorded on a Bruker Avance^TM^ instrument (600 MHz). The optical rotation was measured using the Polarymeter polAAr 31.

### 3.2. Synthesis of 1-aminoethanephosphonic Acid

The substrate was synthesized according to the standard three-component reaction of benzyl carbamate (0.05 M), triphenylphosphite (0.05 M) and acetaldehyde (0.07 M) followed by hydrolysis in concentrated hydrochloric acid [42]. The product was obtained with the yield of 90%. NMR data: ^31^P- NMR: δ (ppm) 16.0; ^1^H-NMR δ (ppm) 1.46 (dd, *J* = 7.1, 16.5, CH*CH_3_*), 3.6 (m, *CH*CH_3_). 

### 3.3. Microorganism Cultivation

The strains of *Beauveria bassiana* (DSM 875), *Penicillium crustosum* (DSM 62837), *Aspergillus fumigatus* (individual collection of the University of Pavia, Italy) and *Cladosporium herbarum* (DSM 63422) were cultivated with shaking (100 rpm) at room temperature in a standard potato dextrose liquid medium (DSMZ Medium 129, 100 mL) in 250 mL cultivation flasks, until the mid-log growth phase was reached (for 3-4 days). *Cuninghamella echinulata* strain (DSM 1905) was cultivated in the standard malt extract peptone liquid medium (DSMZ Medium 90) under the same conditions as the other microorganisms. After the cultivation time the biomass was separated by centrifugation (4,000 rpm/10 min) what allowed obtaining about 8 g of wet cells from every cultivation flask. After that, depending on the down stream, cells were used directly or preincubated for the next 24 h under starvation conditions and then used as biocatalysts. 

### 3.4. Biotransformation: General Procedure

Biotransformation (50 mM of the substrate, 8 g wet cells) was carried out (in three simultaneous experiments for every run) in 50 mL of phosphate buffer (0.017 M, pH 6.11) for varying periods of time (1-5 days) with shaking at 250 rpm. Cells were then removed by centrifugation (4,000 rpm, 10 min) and the supernatant was evaporated. Composition of the product was analyzed by means of NMR spectroscopy.

### 3.5. Derivatization of Enantiomerically Enriched 1-aminoethanephosphonic Acid

The tosyl derivative was prepared by heating a mixture of product (30 mg) in 0.4 M phosphate buffer (pH 11, 10 mL) with TsCl (135 mg) in acetonitrile (10 mL) at 50 °C for 30 min [43]. Then the mixture was extracted twice with ethyl acetate, followed by evaporation of volatile components and NMR analysis was performed.

*^31^P-NMR assignments*: NMR spectra were recorded on a Bruker Avance^TM^ instrument operating at 243.1 MHz; measurements were made in deuterium oxide at a temperature of 300K. 

*Determination of product optical purity: enantiomeric excess assignments*: Optical purity of the product and the enantiomeric excess were evaluated using a ^31^P-NMR technique. In order to achieve separation of enantiomers of 1-aminoethanephosphonic acid by the use of cyclodextrin, this phosphonate has to be derivatized with *p*-toluenesulfonyl chloride. Complexation of the aromatic ring of *p*-toluenesulfonate by cyclodextin results in enantiodifferentiation in the ^31^P spectra. The underivatized aminophosphonic acid is not complexed by cyclodextrin [44]. ^31^P-NMR spectra were recorded with addition of α-cyclodextrin/tosylated 1-aminoethanephosphonic acid (2:1 w/w) and pH of the samples was set at 10-11. 

*Determination of the absolute configuration of the products*: Absolute configuration was determined by measurements of the optical rotation (in 1 M NaOH, c=1) after the tosyl group removed by acidic hydrolysis with concentrated HCl. Representative results were as follows: in the case of (A) - Figure 1, [α]_578_ = −6.7 (42% e.e) for *R* enantiomer; in the case (B) - Figure 1, [α]_578_ = +4.4 (25% e.e). These results are in a very good agreement with the literature data for pure enantiomers, which are as follows: [α]_578_= −16 for the *R* enantiomer and + 17 for the *S* one [37]. 

## 4. Conclusions

Although of preliminary nature, our experiments have indicated that the use of biodegradative potential of fungi may serve as a mean of separation of chiral phosphonic acids. 
